# Molecular Evolution of the *Ovgp1* Gene in the Subfamily Murinae

**DOI:** 10.3390/ani15010055

**Published:** 2024-12-29

**Authors:** Miriam Balastegui-Alarcón, Carla Moros-Nicolás, José Ballesta, Mª José Izquierdo-Rico, Pascale Chevret, Manuel Avilés

**Affiliations:** 1 Departamento de Biología Celular e Histología, Facultad de Medicina y de Enfermería, Universidad de Murcia, 30120 Murcia, Spain; miriam.balastegui@um.es (M.B.-A.); carla.moros@um.es (C.M.-N.); ballesta@um.es (J.B.); mjoseir@um.es (M.J.I.-R.); 2 Instituto Murciano de Investigación Biosanitaria Pascual Parrilla (IMIB), 30120 Murcia, Spain; 3 Laboratoire de Biométrie et Biologie Evolutive, UMR 5558, CNRS, Université Claude Bernard Lyon 1, Université de Lyon, 69100 Villeurbanne, France

**Keywords:** *Ovgp1*, oviduct, chitinases, glycosyl hydrolase 18 family, phylogeny

## Abstract

OGP, encoded by the *Ovgp1* gene, is the major non-serum oviductal protein in most mammals. In the genome of *Rattus norvegicus*, *Ovgp1* has been identified as a pseudogene. However, *Mus musculus* presents a functional gene. As the rat and the mouse belong to the subfamily Murinae, *Ovgp1* has probably been lost after their divergence. This study aims to determine when the pseudogenization event occurred and which proteins could replace its function. To attain that, the potential expression of members belonging to the GH18 family is investigated in the rat oviduct by means of molecular and proteomic analyses. Specific *Ovgp1* regions are sequenced in different murine rodent species. The analysis reveals the presence of stop codons only in some species of the Rattini tribe, suggesting that the majority of the murine species present a functional gene. Thus, the pseudogenization of *Ovgp1* could be dated back to around 10 Mya, after the divergence of the Rattini tribe. The expression of several genes and proteins of the GH18 family, such as *Chia*, *Chit1*, *Chi3l1*, and *Chid1*, are detected in the rat oviduct. This study opens the door for further research on GH18 family proteins that mimic the OGP functions in species where *Ovgp1* is pseudogenized.

## Simple Summary

Different studies have reported that some genes involved in reproduction show a sequence divergence among mammalian species. Over the course of evolution, there has been a gradual loss of genes encoding oviductal proteins undergoing particularly rapid evolution. This fact suggests a diversification of their function and role in different species, with a possible impact on the speciation process. In this study, the evolution of *Ovgp1*, a gene coding for the oviduct-specific glycoprotein (OGP) that is part of the glycosyl hydrolase 18 family (GH18), is analyzed by phylogenetic analysis in several rodents of the subfamily Murinae. Within this subfamily, *Ovgp1* is a functional gene in the mouse, whereas *Ovgp1* is pseudogenized in the rat. This analysis allows us to determine when this pseudogenization event occurred and which other species of the subfamily are affected. Due to the potential reproductive function of OGP in other mammalian species, we study possible candidates to supply its function in rats. Using molecular and proteomic techniques, we determine the expression and oviductal presence of four members of the GH18 family: *Chia*, *Chit1*, *Chi3l1*, and *Chid1.*

## 1. Introduction

Mammalian fertilization is a complex process that involves two cells, the sperm and the oocyte, and it takes place in the lumen of a specialized conduct called oviduct or fallopian tube in the human species. The gametes complete their maturation in the oviduct, and, after fertilization, the first divisions of the zygote are produced in this conduct. It has been demonstrated that the oviductal secretion and environment play a crucial role in gamete maturation, fertilization, and early embryo division. The composition of the oviductal fluid (OF) is complex and entails components secreted by epithelial cells and exudates from the blood plasma [1,2,3,4,5,6].

Albumin and OGP are the main protein components of OF. Albumin is produced in the liver and is transferred to the lumen of the oviduct via blood transudation. OGP is produced by non-ciliated cells, and it is a member of the GH18 family, which includes proteins with chitin-hydrolyzing properties; however, no enzymatic activity has been described for this oviductal protein [7,8,9]. OGP has been detected in several mammals, including monotremes [10], marsupials [11], and placentals, including humans [12,13]. However, it has been reported that, in some species such as rats and megabats, *Ovgp1* is a pseudogene, meaning that the OGP protein is not expressed in the oviduct [14,15,16]. The loss of *Ovgp1* is not rare, as gene loss events have occurred throughout the evolutionary history of mammals [17,18,19]. Moreover, it is well known that genes related to reproduction evolve faster than genes expressed in other tissues [20,21,22,23]; examples include ZP genes, such as *Zp2* and *Zp3*, sperm genes, acrosin, and seminal plasma proteins [24,25]. Rapid evolution can also be associated with loss of functionality, sometimes leading to pseudogenization [26,27,28] or to adaptive evolution induced by natural selection [21]. This rapid evolution can be species-specific, and it could even play a role in speciation, as it has been described for CD9 in the oocyte and IZUMO1 in the sperm [29]. Oviductal proteins are not an exception [16]. The fact that *Ovgp1* is not expressed in some species and that *Ovgp1* gene-null mice have apparently normal fertility [30] suggests that this glycoprotein is not essential for fertilization in some species or that there are other proteins with a redundant function as described in sperm acrosomal proteases [31,32].

The aim of the present study is to provide new information about the *Ovgp1* DNA sequence in a high number of rodents to investigate if the pseudogenization of *Ovgp*1 is restricted to the Rattini tribe of the subfamily Murinae. Moreover, we investigate if other genes of the GH18 family are expressed in the rat oviduct. All these results provide information about the specific proteins of the GH18 family in the oviduct. Further studies are required to clarify the role of the different oviductal chitinases during fertilization and preimplantation embryo development.

## 2. Materials and Methods

**Ethanol-preserved material**

Ethanol-preserved tissue samples were obtained from the tissue collection of the Institut des Sciences de l’Evolution and the CBGP—Small mammal Collection of Montpellier (France).

**Samples obtained from animals in vivo**

Fifteen fertile female rats (*Rattus norvegicus*) were used in this study. Two experimental groups were designed: (1) post-ovulatory females (presence of cumulus–oocyte complexes) and (2) females on day 3 post-fertilization (92 h post natural mating, morula stage). A total of three animals per experiment were used for each experimental group. Animals were kept under standard laboratory rodent conditions in an environmentally controlled room with a 14 h light:10 h darkness photoperiod under constant temperature and relative humidity conditions. Animals were provided with food (Harlan Ibérica, Barcelona, Spain) and water, both available ad libitum. All animals were sacrificed by CO_2_ overdose. For synchronization, females were subjected to hormonal treatment by intraperitoneal (i.p.) injection of 15 IU of pregnant mare serum gonadotropin (PMSG, Foligon 1000UI, MSD Animal Health, Salamanca, Spain), followed, 48 h later, by i.p. injection of 15 IU of human chorionic gonadotropin (hCG, Veterin corion 750 IU, DFV^®^, Barcelona, Spain). To obtain oviducts containing cumulus–oocyte complexes, rats were sacrificed 17 h after hCG injection (day 1 at 09:00 h). To obtain the oviducts on day 3 after fertilization, females were crossed with males immediately after hCG injection and sacrificed 92 h later (day 4 at 12:00 h). The regularity of the estrous cycle of the females was determined prior to the hormonal synchronization treatment by daily exfoliative vaginal cytology, covering two complete estrous cycles. Immediately after the sacrifice, the oviducts were dissected and the OF and cumulus–oocyte complexes were recovered by oviductal flushing from the infundibulum using 300 µL Dulbecco PBS (Merck KGaA, Darmstadt, Germany). In the case of oviducts on day 3 after fertilization, the OF was recovered using the same oviductal flushing technique. In both experimental groups, the recovered OF was centrifuged at 14,000 rpm for 5 min to separate and preserve the supernatant. The protein concentration in the supernatant samples from both experimental groups was determined using the Bradford colorimetric technique.

Samples were preserved according to the target technique. For oviductal RNA extraction, oviducts were preserved at −80 °C in the RNAlater™ reagent (Merck KGaA, Darmstadt, Germany) until processing. For the proteomic study, the OF was preserved at −20 °C until processing. For the preparation of the paraffin blocks, the oviducts were fixed in 4% formaldehyde at room temperature for 24 h, then tissue samples were dehydrated and embedded in Paraplast Plus Paraffin (Merck KGaA, Darmstadt, Germany).

### 2.1. Phylogenetic Analysis

#### 2.1.1. Genomic DNA Extraction

gDNA was extracted from the ethanol-preserved tissues of 20 species belonging to the subfamily Murinae (Table 1), using the nucleospin tissue kit (Macherey-Nagel, Düren, Germany) according to the manufacturer’s recommendations.

#### 2.1.2. PCR Primer Design

The *Mus musculus Ovgp1* gDNA sequence was 13,622 bp (ENSMUSG00000074340) and contained 11 exons (NM_007696.2). Using the BLAST (Basic Local Alignment Search Tool) program (https://blast.ncbi.nlm.nih.gov, accessed on 10 March 2024) [33], we searched the GenBank [34] and Ensembl [35] databases for matches for the mRNA *Mus musculus* sequence (NM_007696.2) and members of the family Muridae. The sequences of 10 species belonging to this family that were available at the start of the study were selected (Table 1), and multiple alignments were performed with the MUSCLE program in SeaView [36]. Primers were designed in conserved regions. The amplicons of the eight designed primer pairs covered the gDNA regions of the *Ovgp1* gene corresponding to exons 1 to 6 (Table S1; Figure S1).

#### 2.1.3. PCR and Electrophoresis

PCR mixtures of 25 μL were prepared using 20–25 ng gDNA, 0.5 μL of AmpliTaq Gold^®^ polymerase enzyme (Applied Biosystems, Foster City, CA, USA), 2.5 μL of 10X buffer, 2 μL of dNTP (2.5 mM), 2 μL of MgCl2 (25 mM), 1.25 μL of each primer (10 μM), and 8.75 μL of water. A polymerase chain reaction was performed using an initial denaturation cycle of 3 min at 94 °C, followed by 40 reactions of 30 s at 90 °C, 15 s at annealing temperature (depending on the primers), and then 90 s at 72 °C. The final extension time was 15 min at 72 °C. PCR products were analyzed by electrophoresis on 1.5% agarose gels with loading dye 6X (Thermo Fisher Scientific, Waltham, MA, USA) at 100 V for 20 min. A GeneRuler 1 kb DNA Ladder (Thermo Scientific, Waltham, MA, USA) was used as a reference DNA marker and GelRed (GelRed^®^ Nucleic Acid Stain, Biotium, Fremont, CA, USA) was used as a DNA marker. The amplified DNA products were visualized using a transilluminator (Alphalmager MINI, Cell Bio-sciences, Santa Clara, CA, USA). PCR products showing a single band and a DNA concentration > 10 ng/μL were sequenced at Microsynth France SAS (Vaux-en-Velin, Auvergne-Rhône-Alpes, France).

The new sequences were deposited at the ’European Nucleotide Archive’ (https://www.ebi.ac.uk/ena/browser/home, accessed on 12 December 2024) under the accession numbers OZ209766-OZ209785.

#### 2.1.4. Phylogenetic Tree Construction and Genetic Distance Analysis

A multiple alignment covering exons 1 to 6 in 42 species of the subfamily Murinae was obtained using the SeaView program [36]. Sequences of Deomyinae and Gerbillinae were also included as outgroups in the multiple alignment (Table 1). Only the exonic areas (626 bp) were used for the phylogenetic analysis. Phylogenies were estimated using the maximum likelihood method in the PhyML 3.1 program [37] and the Bayesian inference in the MrBayes 3.2 program [38]. We used the online version of PhyML (http://www.atgc-montpellier.fr/phyml/, accessed on 24 September 2024) and the best-fitting evolutionary model was determined by SMS [39]. For the Bayesian inference, the MrBayes program used the parameter Nst = mixed that allows for the sampling of different substitution models. The robustness of the nodes was estimated by using 1000 bootstraps in the PhyML program and subsequent tests in the MrBayes program. For the Bayesian analysis, the number of generations was set to 2,000,000, with a tree sampled every 500 generations. The burn-in (10%) was determined graphically with Tracer v.1.7 [40], checking that the effective sample sizes were well over 200 and that the average standard deviation of the split frequencies remained < 0.05 after the burn-in threshold. Figtree v.1.4 [41] was used to visualize the phylogenetic tree.

### 2.2. Rattus Norvegicus Oviductal Analysis

#### 2.2.1. Oviductal RNA Extraction and In Vitro cDNA Synthesis

The oviductal RNA was extracted using the RNAqueous Phenol-free Total RNA Isolation kit (Invitrogen, Carlsbad, CA, USA) following the manufacturer’s instructions. RNA quality and concentration was evaluated spectrophotometrically (NanoDrop ND-1000, Thermo Fisher Scientific, Waltham, MA, USA). cDNA synthesis was performed by retrotranscription using the QuantiTect Reverse Transcription kit (Qiagen, Hilden, Germany) following the manufacturer’s recommendations.

#### 2.2.2. RT-qPCR Amplification

RT-qPCR amplifications were performed using oviductal cDNA as the template. Rat-specific SYBR green primers were custom-designed for *Chia* (NM_207586), *Chit1* (NM_001079689, NM_001270847, NM_001270846, NM_001270848), *Chit3l1* (NM_001309820, NM_053560), *Chid1* (NM_001047854, NM_001421299, NM_001421300), *Actb1* (NM 031144), and *Hprt1*(NM_012583) sequences obtained from Merck KGaA (Darmstadt, Germany) (Table S2). These genes, members of the GH18 family, were selected in light of preliminary RNA-seq results from rat oviducts obtained by our group (unpublished data). A quantitative PCR analysis was performed using the Genomic Platform at the IMIB-Pascual Parrilla using SYBR Premix Ex Taq II (Tli RNaseH Plus, Takara Bio, Saint-Germain-en-Laye, France) in the QuantStudio 5 Real-Time PCR System (Applied Biosystems, Foster City, CA, USA). Each PCR reaction was conducted in a 5 μL volume with a primer concentration of 450 nM. The cycling conditions consisted of an initial denaturation step at 95 °C for 30 s, followed by 40 cycles of denaturation at 95 °C for 5 s, and annealing/extension at 60 °C for 34 s. All samples were run in triplicates. A gene expression analysis was performed using the 2^−ΔΔCt^ method, where ΔCt represents the difference between the threshold cycle of a given target cDNA and an endogenous reference gene (*Actb1* and *Hprt1*).

#### 2.2.3. Proteomic Analysis

OF samples were digested using the following standard procedure. Samples were prepared in a final volume of 100 µL of 50 mM ammonium bicarbonate buffer pH 8.0 with 0.01% ProteaseMax (Promega, Madison, WI, USA). This surfactant enhances trypsin digestion. Protein samples were reduced by adding 10 mM DTT at 56 °C for 20 min. Then, samples were alkylated by adding 25 mM iodoacetamide for 30 min at room temperature in the dark. Finally, digestion was performed by adding Trypsin Gold Proteomics Grade (Promega, Madison, WI, USA) at an approximate ratio of 1:100 *w*/*w* over 16 h at 37 °C. The reaction was stopped with 0.1% formic acid and filtered through 0.2 µm filters. Finally, samples were dried using an Eppendorf vacuum concentrator, model 5301.

The separation and analysis of the tryptic digests of the samples were performed with an HPLC-MS/MS system consisting of an Agilent 1290 Infinity II Series HPLC (Agilent Technologies, Santa Clara, CA, USA) equipped with an automated multisampler module and a high-speed binary pump and connected to an Agilent 6550 Q-TOF mass spectrometer (Agilent Technologies, Santa Clara, CA, USA) using an Agilent jet stream dual electrospray (AJS-Dual ESI) interface. The experimental parameters for HPLC and Q-TOF were set using the MassHunter Workstation Data Acquisition software (Agilent Technologies, version B.08.00).

Dry samples from trypsin digestion were resuspended in 20 µL of buffer A, consisting of water/acetonitrile/formic acid (94.9:5:0.1). The sample was injected into an Agilent AdvanceBio Peptide Mapping HPLC column (2.7 µm, 100 × 2.1 mm, Agilent Technologies), thermostatted at 50 °C, at a flow rate of 0.4 mL/min. This column is suitable for peptide separation and analysis. After the injection, the column was washed with buffer A for 3 min and the digested peptides were eluted using a linear gradient 0–40% B (buffer B: water/acetonitrile/formic acid, 10:89.9:0.1) for 40 min followed by a linear gradient 40–95% B for 8 min. The 95% B was maintained for 3 min. Finally, the column was equilibrated under the initial conditions for 6 min before every injection.

The mass spectrometer was operated in the positive mode. The nebulizer gas pressure was set to 35 psi, whereas the drying gas flow was set to 14 L/min at a temperature of 300 °C, and the sheath gas flow was set to 11 L/min at a temperature of 250 °C. The capillary spray, nozzle, fragmentor, and octopole RF Vpp voltages were 3500 V, 100 V, 360 V, and 750 V, respectively. Profile data were acquired for both MS and MS/MS scans in the extended dynamic range mode at 4 GHz. The MS and MS/MS mass range was 50–1700 *m*/*z* and the scan rates were 8 spectra/sec for MS and 3 spectra/sec for MS/MS. The auto MS/MS mode was used with precursor selection by abundance and a maximum of 20 precursors selected per cycle. A ramped collision energy was used with a slope of 3.68 and an offset of −4.28. The same ion was rejected after two consecutive spectra.

Data processing and analysis was performed using the Spectrum Mill MS Proteomics Workbench software (Rev B.06.00.201, Agilent Technologies, Santa Clara, CA, USA).

Briefly, the raw data were extracted under default conditions as follows: unmodified or carbamidomethylated cysteines, [MH]+50–10,000 *m*/*z*, a maximum precursor charge of +5, a minimum signal-to-noise MS (S/N) of 25, and identification of 12C signals.

The MS/MS search against the appropriate and updated protein database was performed with the following criteria: (i) a variable modification search mode (carbamidomethylated cysteines, STY phosphorylation, oxidized methionine, and N-terminal glutamine conversion to pyroglutamic acid), (ii) tryptic digestion with five maximum missed cleavages, (iii) ESI-Q-TOF instrument, (iv) a minimum matched peak intensity of 50%, (v) a maximum ambiguous precursor charge of +5, (vi) monoisotopic masses, (vii) a peptide precursor mass tolerance of 20 ppm, (viii) a product ion mass tolerance of 50 ppm, and (ix) calculation of reversed database scores. The validation of peptide and protein data was performed using auto thresholds.

#### 2.2.4. Immunohistochemistry

Chitinase domain-containing protein 1 (CHID1) and chitinase-3-like protein 1 (CHI3L1) expression was immunohistochemically analyzed in the oviduct. Sections from the postovulatory oviduct (5 μm) were placed on Superfrost Plus microscope slides (Menzel, Braunschweig, Germany), which were then deparaffined in xylene and rehydrated through a descending ethanol series followed by distilled water. Antigen retrieval was performed by boiling the sections in a citrate buffer pH 6 antigen retriever (Merck KGaA, Darmstadt, Germany) for 30 min. To block endogenous peroxidase activity, the sections were incubated for 30 min in a solution of PBS with 1% H_2_O_2_. After washing with TBS-Tween, background noise blocking was performed by incubating the sections with 2.5% normal horse serum (Vector laboratories, Newark, CA, USA) for 20 min at 37 °C. After rinsing with TBS (Merck KGaA, Germany), the slides were incubated individually with a polyclonal anti-CHID1 primary antibody, with a dilution of 1:50 (HPA039374, Merck KGaA, Germany), and a polyclonal anti-CHI3L1 primary antibody, with a dilution of 1:2500 (PA5-37357, Thermo Fisher, Waltham, MA, USA), overnight at 4 °C in a humid chamber. Then, samples were tempered at 37 °C for 10 min, washed for 3 min with TBS-Tween, incubated for 30 min with a horseradish peroxidase (HRP)-conjugated rabbit anti-immunoglobulin G secondary antibody polymer (MP-7801, Vector Laboratories, Susteren, The Netherlands) at 37 °C, and washed twice for 2 min with TBS-Tween.

The immunoreaction was revealed with 0.5 mg/mL solution of 3,3′-diaminobenzidine in TBS (Merck KGaA, Germany), counterstained with hematoxylin, dehydrated, cleared through xylene, and mounted in dibutyl phthalate xylene (DPX) (BDH Prolabo, VWR International Ltd., Leicestershire, UK). Negative controls were performed following the same procedure without incubation with the primary antibodies.

Visualization of the histological sections was performed with an Olympus BX51 light microscope (Olympus, Allentown, PA, USA), and micrographs were taken with an attached Olympus DP 25 camera (Olympus, Allentown, PA, USA). Images were analyzed using the Cell D image analysis software (Olympus, Allentown, PA, USA).

## 3. Results

### 3.1. Phylogenetic Analysis of the Ovgp1 Gene in the Subfamily Murinae

The genomic DNA region encompassing exons 1–6 of the *Ovgp1* gene was amplified and sequenced in 20 species of the subfamily Murinae (Table 1). Depending on the species, 2 to 6 exons were sequenced (Figure S1).

**Figure 1:**
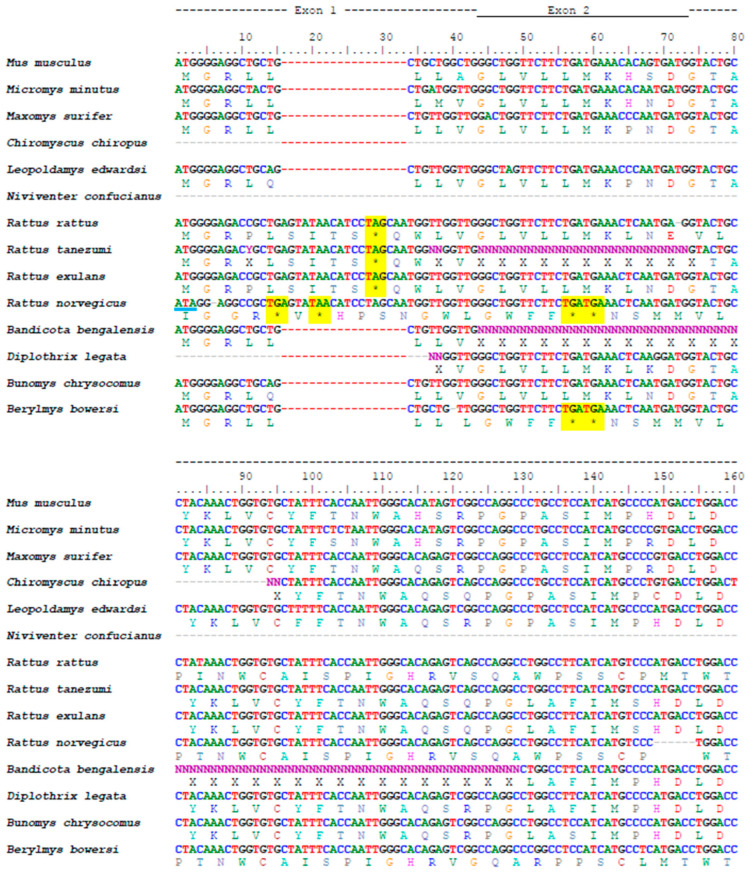

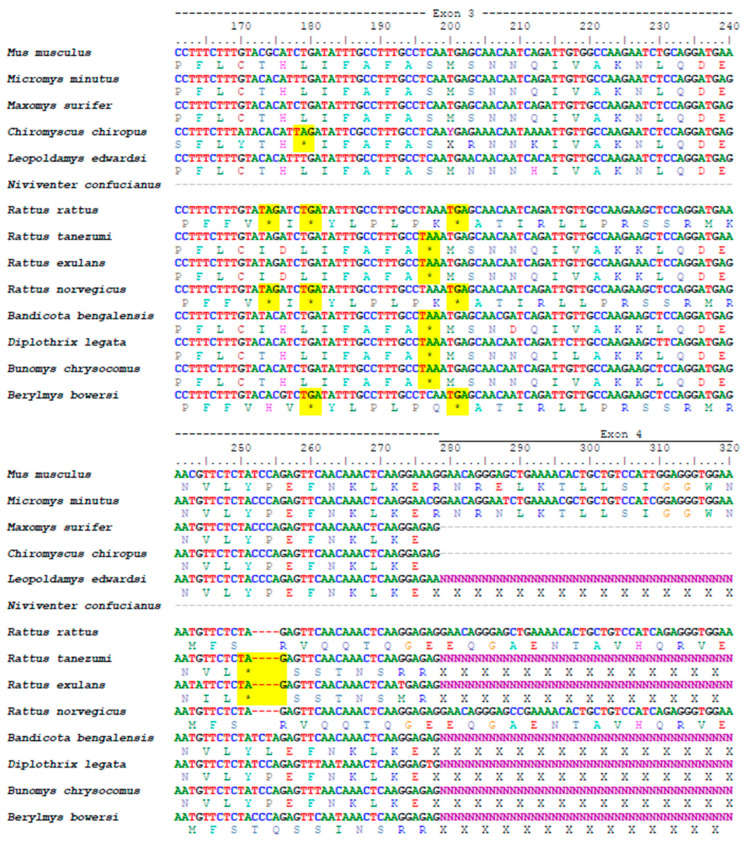

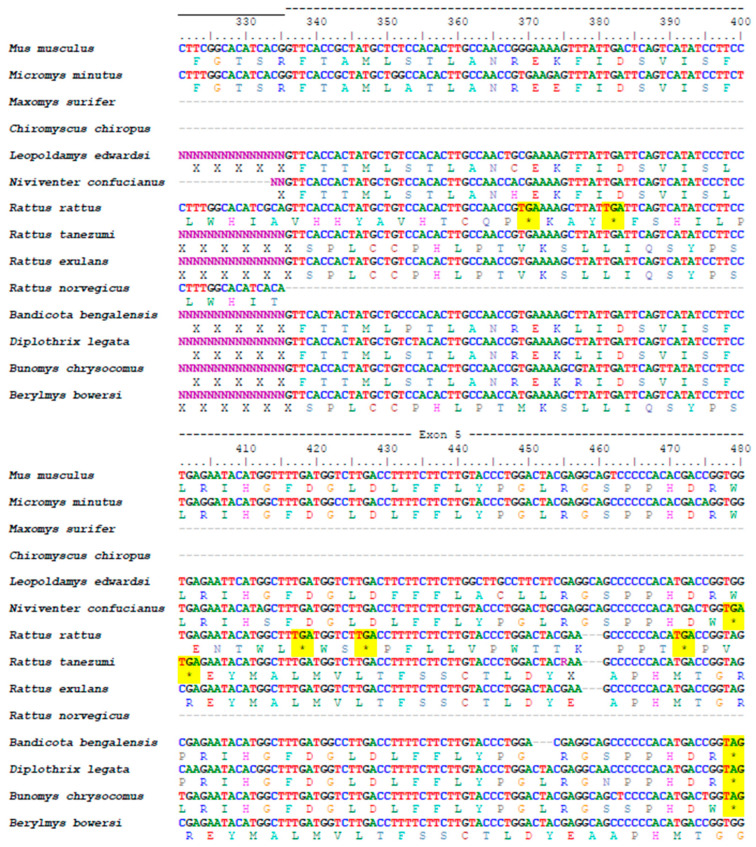

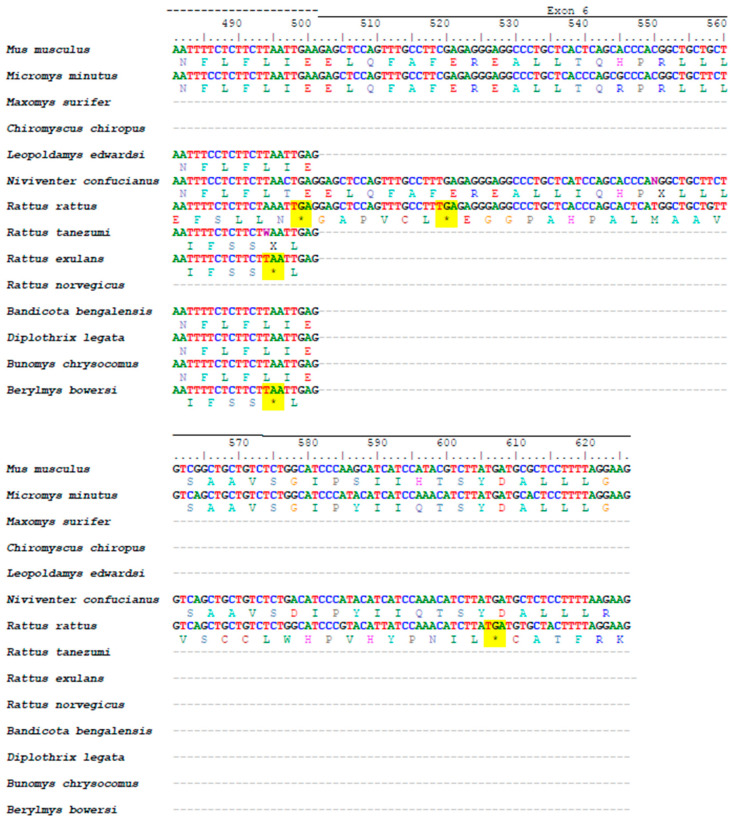
Multiple alignment of nucleotide and amino acid sequences of the Rattini species analyzed. The functional sequence of *Mus musculus* was added for comparison. The stop codons are highlighted in yellow. The letter “N” in the nucleotide sequence and the letter “X” in the amino acid sequence appear when the base or amino acid, respectively, could not be determined. The letter “Y” in the nucleotide sequence indicates that the nucleotide at that position may be either C or T. The presence of the symbol “-” indicates the absence of a sequence due to a lack of sequencing or an actual deletion. The symbol “*” indicates a stop codon.

**Figure 2:**
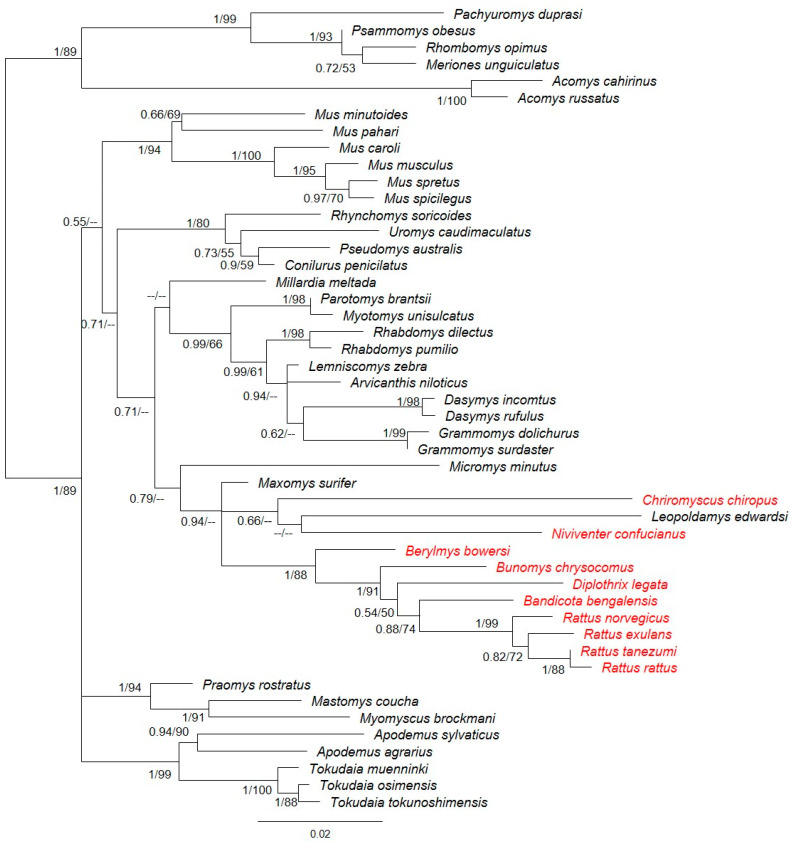
Phylogenetic relationships with respect to the *Ovgp1* gene in different species of murid rodents. The scale indicates the number of substitutions per site. Bootstrap support and posterior probabilities are indicated for each node. “- -” indicates that these nodes are not supported by maximum likelihood or Bayesian methods. Species in which stop codons were observed are colored in red (all belong to the Rattini tribe).

The multiple alignment of all the analyzed species belonging to the Rattini tribe (Figure 1) showed that the insertion in exon 1 previously described in *Rattus norvegicus* (Tian et al., 2009 [15]) is also present in *Rattus exulans*, *Rattus rattus*, and *Rattus tanezumi*. In these species, the deletion of four bases in position 251 in exon 3 also occurred. However, *Rattus norvegicus* is the only species that presents a mutation in the initiation codon (ATG → ATA) and a deletion of one base in position 6 in exon 1. These indels cause the reading frame to change, triggering the formation of premature stop codons between exons 1 and 6. Six other genera of the Rattini tribe also showed premature stop codons between exons 1 and 6: *Niviventer*, *Chiromyscus*, *Berylmys*, *Bandicota*, *Bunomys*, and *Diplothrix*. Of these, *Berylmys bowersi* exhibited the deletion of one base in position 22 in exon 1. The position of the stop codons present in each species is listed in Table 2. The multiple alignment of the non-Rattini murid species analyzed without premature stop codons is shown in Figure S2. The phylogenetic tree (Figure 2) confirms the monophyly of the Rattini tribe. Pseudogenization was found in the first six exons of the gene in almost all the Rattini species tested, except for *Micromys*, *Maxomys*, and *Leopoldamys*. This result indicates that pseudogenization took place after the divergence of the Rattini tribe and after the divergence of *Micromys*, as we retrieved the full length *Ovgp1* gene for this taxon. Based on our partial sequences, the pseudogenization might have taken place after the divergence of *Maxomys* at the base of the two other Rattini groups.

### 3.2. The Rat Oviduct Expresses Other Chitinases Besides Ovgp1

Preliminary RNA-seq results obtained by our group (unpublished data) detected the expression of four members of the GH18 family in the rat oviduct, encoded by the *Chia, Chit1, Chi3l1*, and *Chid1* genes. The transcript abundance of these four genes was compared between the two experimental groups, (1) post-ovulatory females containing cumulus–oocyte complexes and (2) females on day 3 post-fertilization, to ascertain whether the expression changed between the two stages. Our results show that there were no significant differences in the relative transcript abundance between the two experimental groups.

*Chia*, *Chit1*, *Chi3l1*, and *Chid1* mRNAs are effectively translated into proteins, as confirmed by the detection by HPLC-MS/MS analysis of several peptides belonging to the proteins encoded by those four genes in the rat OF in both experimental groups analyzed. A total of 11 different peptides belonging to the amino acid sequence of CHID1, 13 peptides of CHI3L1, 10 peptides of chitinase (CHIT1), and seven peptides of acidic mammalian chitinase (CHIA) were detected in the OF from post-ovulatory oviducts (Table 3). A total of nine different peptides belonging to the amino acid sequence of CHID1, seven peptides of CHI3L1, nine peptides of CHIT1, and 11 peptides of CHIA were detected in the OF from oviducts on day 3 post-fertilization (Table 4).

### 3.3. Immunohistochemistry

**Figure 3:**
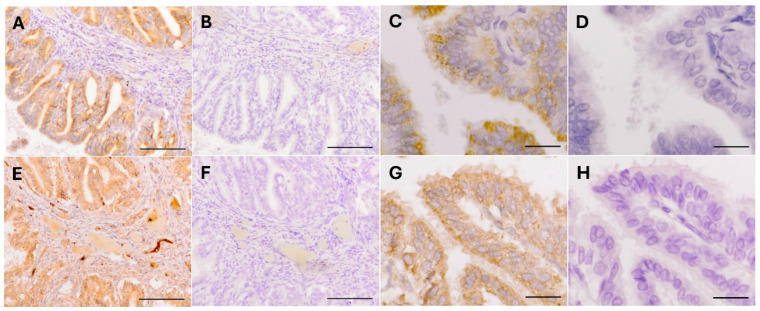
(**A**) Immunohistochemical localization of CHID1 in the post-ovulatory rat oviduct. CHID1 labeling was localized to the oviductal epithelium. Bar = 100 µm. (**B**) No labeling observed in the negative control image (no primary antibody). Bar = 100 µm. (**C**) Higher magnification of the oviductal epithelium showing that the epithelial cells show more intense punctate labeling in the apical region, with fainter labeling in the basal region. Bar = 20 µm. (**D**) No labeling observed in the negative control image (no primary antibody). Bar = 20 µm. (**E**) Immunohistochemical localization of CHI3L1 in the post-ovulatory rat oviduct, with CHI3L1 labeling detected in the subepithelial connective tissue and, more intensely, in the oviductal epithelium. Bar = 100 µm. (**F**) No labeling observed in the negative control image (no primary antibody). Bar = 100 µm. (**G**) Higher magnification of the oviductal epithelium revealing that the epithelial cells show intense labeling in the apical zone and fainter labeling in the basal zone. Bar = 20 µm. (**H**) No labeling observed in the negative control (without primary antibody). Bar = 20 µm.

The oviductal location of CHID1 and CHI3L1 proteins was determined in rat oviduct tissues from the post-ovulatory phase. Immunolabeling for CHID1 and CHI3L1 proteins was localized to the oviductal epithelium. In the case of CHI3L1, labeling was also observed in the subepithelial connective tissue. At the level of the oviductal epithelium, in the case of both CHID1 and CHI3L1, intense punctate labeling was observed in the apical zone of the epithelial cells and a fainter labeling was detected in the basal zone (Figure 3).

## 4. Discussion

### 4.1. Molecular Evolution of the Ovgp1 Gene in the Subfamily Murinae

Previous studies have identified numerous proteins in the OF from several mammalian species [42,43,44,45,46,47]. OGP is one of the most abundant proteins in OF with important functions in reproductive events. This protein has been shown to play a role in the fertilization process of goats [48], pigs [49], cows [50], buffalos [51], hamsters [52,53,54], and humans [55,56]. It attaches to the zona pellucida (ZP) and the spermatozoa and improves in vitro fertilization and embryo development. Interestingly, knock out (KO) in the mouse (*Mus musculus*), a species belonging to the family Muridae and the large superfamily Muroidea, does not impact female reproduction [30], implying that, at least in the *Mus* genus, *Ovgp1* is not essential for fertility [57,58]. However, its deletion (KO) in the golden hamster (*Mesocricetus auratus*), a myomorphic rodent that is part of the family Cricetidae and, like the mouse, belongs to the large superfamily Muroidea, leads to female infertility [57,58]. Furthermore, within the same order Rodentia*,* to which both species belong (mouse and golden hamster), we find the rat (*Rattus norvegicus*), a member of the subfamily Murinae like the mouse, where *Ovgp1* is pseudogenized in its genome [15]. Pseudogenization of *Ovgp1* has also been observed in the megabat [16], belonging to the order Chiroptera, superfamily Pteropodoidea, and family Pteropodidae. The *Ovgp1* gene has been described to undergo rapid adaptive evolution [59], responsible for the divergence of this protein in mammalian species. Within the *Ovgp1* family, positive selection has been demonstrated in the dog [16], while duplication has been demonstrated in the cow, sheep, and sow [16].

Our phylogenetic study of the *Ovgp1* gene within the subfamily Murinae, to which mice and rats belong, provides interesting insights into its evolution. The results indicate that pseudogenization within the subfamily affects not only the rat (*Rattus norvegicus*), as previously demonstrated [15], but also other species within the genus *Rattus: R. exulans, R. rattus*, and *R. tanezumi*. In addition, the genera *Chiromyscus, Niviventer, Berylmys, Bandicota, Bunomys*, and *Diplothrix,* which, like *Rattus*, belong to the Rattini tribe, also present premature stop codons between exons 1 and 6. The remaining seven tribes included in our study appear to have a functional *Ovgp1*. Based on these results, it appears that pseudogenization only occurred within the Rattini tribe, that is, after the split between the Rattini and the other Murinae. As we were able to obtain, from Genbank, a full-length sequence of *Ovgp1* for *Micromys*, the pseudogenization occurred after the divergence of *Micromys* and the other Rattini. For *Maxomys* and *Leopoldamys*, we were only able to obtain partial sequences (six exons out of 11) where we found no evidence of a stop codon. It would be interesting to complete the sequences of these two taxa to confirm the absence of stop codons.

The extensive taxonomic sampling conducted within the subfamily, particularly within the Rattini tribe (with ten genera represented), enabled us to ascertain the approximate date of the loss of *Ovgp1*. Previous studies have reported that the Rattini tribe diverged from the other Murinae between 11 Mya and 12 Mya [60,61]. Within the Rattini tribe, *Micromys* was the first genus to diverge 10 Mya; as *Micromys* species have a full-length *Ovgp1* gene without a stop codon, pseudogenization was likely more recent. It would be interesting to investigate the presence of a functional *Ovgp1* gene in other species of *Maxomys* and in the *Dacnomys* division, as well as in the *Echiothrix* division that was not included in our study. This study provides evidence of a probable *Ovgp1* pseudogenization process in the Rattini tribe, such process occurring after the divergence of *Maxomys* from the other Rattini. A similar process has also been observed in other reproductive proteins such as ZP proteins that surround the oocyte [62,63,64,65,66].

### 4.2. Members of the GH18 Family of Chitinases with Expression in the Rat Oviduct

Previous studies have shown that OGP plays a potential role in the reproductive process of mammals. However, contradictory results have been obtained when comparing the effect of *Ovgp1* loss in mice and hamsters. The KO mouse is fertile [30]; however, the deletion of *Ovgp1* (KO) in the golden hamster (*Mesocricetus auratus*) leads to female infertility [57,58]. Based on these premises, it would be interesting to determine whether, in species in which *Ovgp1* has undergone pseudogenization and, therefore, no functional OGP protein is present, there are other proteins present in the OF that could perform a similar function.

OGP is a protein belonging to the GH18 family; however, this protein does not undertake chitinase activity due to the lack of an essential glutamic acid residue present in the active sites of the chitinases [67,68]. A phylogenetic analysis of the members of the GH18 family has shown that duplication and divergence events lead to the emergence of multiple forms of active chitinases and inactive chitinase-like proteins. OGP has a close evolutionary relationship with mammalian acid chitinases (AMCases) [51]. Mammals produce two chitinases belonging to the GH18 family, CHIT1 and CHIA [69]. Additionally, mammals encode multiple chitinase-like proteins that lack chitinolytic activity [70,71,72,73]. A previous transcriptomic analysis of the mouse oviduct using RNA-seq technology has demonstrated the expression of proteins of the GH18 family [74].

Our molecular analysis of the rat oviduct and our proteomic analysis of the OF have demonstrated oviductal expression of four members of the GH18 family: *Chia*, *Chit1*, *Chi3l1*, and *Chid1*. Differences between the two different stages analyzed were not detected. This result is in accordance with results recently obtained from the mouse oviductal transcriptome during the estrous cycle, suggesting that the lack of differences is probably due to the short duration of the cycle in this species and others, such as the rat used in this study, compared to other species such as humans [74].

*Chid1* is expressed only in humans and rodent species, and it is not essential for fertilization in mice, as demonstrated by the generation of a KO model [75]. In the human fallopian tube, the presence of the CHID1 in the oviductal epithelium has also been detected [76]. In our study, a strong immunostaining was found in the rat oviductal epithelium. The protein was also detected in the OF by proteomic analysis, confirming the presence of this protein in the oviductal milieu. However, this enzyme is not expected to be present in the OF, as it has been demonstrated by its presence in the lysosome organelles [75]. Then, its presence could be ascribed to a different secretion mechanism or to the exfoliation of the epithelial cells. Another possibility is that this enzyme could be secreted, as it has been previously reported, by macrophages and tumor cell lines of epithelial origin [77]. Moreover, the presence in the OF could also be attributed to the presence of white blood cells, as it has been previously reported in the bronchoalveolar lavage [77]. White blood cells have been previously detected in the oviductal lumen [78,79].

*Chit1* shows a high degree of similarity among species, being highly conserved among humans, chimpanzee, mouse, and rat species [80]. It is a non-essential protein for fertilization in mice, as demonstrated by the generation of the KO model [81]. Previously, it has been shown that this protein is secreted by macrophages and transfected COS-1 cells and is present in the human plasma [82,83]. In our study, the protein was detected in the OF by proteomic analysis, confirming the presence of this protein in the oviductal milieu and strongly suggesting its secretion by the oviductal epithelium.

CHI3L1 is a glycoprotein expressed and secreted by several cells, including macrophages, neutrophils, and secretory cells of the fallopian tube [76]. This protein plays a role in cell differentiation and proliferation, immune response, and inflammation [84]. The generation of the KO model in the mouse shows that this protein is not essential for fertilization in this species [85]. In this study, the protein was detected in the OF by proteomic analysis and in the oviduct by immunohistochemistry, suggesting its secretion by the oviductal epithelium. This result is compatible with the secretion of the protein as described previously as a result of its presence in the human colostrum [86].

CHIA is a mammalian chitinase secreted by the respiratory epithelium, salivary glands, and stomach [87,88,89,90,91]. The creation of female mice with a deletion of this gene has shown that fertility is not affected in this species [85]. A low immunoreactivity has been detected in the human fallopian tube [76]. In this work, CHIA was detected in the OF by proteomic analysis.

In conclusion, the mouse KO models demonstrate that these proteins belonging to the GH18 family are not indispensable for fertility purposes. This finding may be attributed to a redundant function, with the elimination of only one of these proteins exerting no discernible impact. Further studies, including the generation of multiple KO models, would provide more precise information on the role played by the GH18 family of proteins, as it has been previously reported for acrosomal proteases in sperm [92].

## 5. Conclusions

This study analyzes the pseudogenization of the *Ovgp1* gene in the subfamily Murinae, demonstrating that this process took place exclusively within the Rattini tribe. Furthermore, we demonstrate gene expression and the presence of different proteins belonging to the GH18 family in the rat oviduct. These proteins are present in the OF at the time of fertilization and throughout the subsequent early stages of embryo development. These proteins could be implicated in different roles that remain to be clarified. They may perform a role similar to that of OGP, a phenomenon which could explain why it is not essential for fertility purposes in mice.

## Figures and Tables

**Table 1:** List of species of the family Muridae used in this study.

Subfamily	Tribe	Genus	Species	Sequence
Murinae	Apodemyini	*Apodemus*	*Apodemus agrarius*	OZ030007
			*Apodemus sylvaticus*	XM_052180919 (NC_067475)
		*Tokudaia*	*Tokudaia muenninki*	BTHS01000002
			*Tokudaia osimensis*	BPMZ01000917
			*Tokudaia tokunoshimensis*	BTHU01000003
	Arvicanthini	*Arvicanthis*	*Arvicanthis niloticus*	XM_034500638 (NC_047661) *
		*Dasymys*	*Dasymys incomtus*	Sequence obtained in this study
			*Dasymys rufulus*	Sequence obtained in this study
		*Grammomys*	*Grammomys dolichurus*	JADRCF010501649
			*Grammomys surdaster*	XM_028755672 (NW_021620880) *
		*Lemniscomys*	*Lemniscomys zebra*	Sequence obtained in this study
		*Rhabdomys*	*Rhabdomys dilectus*	JADRCG010009874
			*Rhabdomys pumilio*	JANHMN010000001
	Hydromyini	*Conilurus*	*Conilurus penicilatus*	Sequence obtained in this study
		*Pseudomys*	*Pseudomys australis*	Sequence obtained in this study
		*Rhynchomys*	*Rhynchomys soricoides*	JADRCH010007518
		*Uromys*	*Uromys caudimaculatus*	CM052704
	Millardini	*Millardia*	*Millardia meltada*	Sequence obtained in this study
	Murini	*Mus*	*Mus caroli*	XM_021159142 (NC_034572) *
			*Mus minutoides*	LR750027
			*Mus musculus*	NM_007696 (ENSMUSG00000074340) *
			*Mus pahari*	XM_021197069 (NC_034593) *
			*Mus spicilegus*	ENSMSIT00000008008 (MUSP714) *
			*Mus spretus*	T0062152 (SPRETEiJ) *
	Otomyini	*Myotomys*	*Myotomys unisulcatus*	Sequence obtained in this study
		*Parotomys*	*Parotomys brantsii*	Sequence obtained in this study
	Praomyini	*Mastomys*	*Mastomys coucha*	XM_031376141 (NW_022196898) *
		*Myomyscus*	*Myomyscus brockmani*	Sequence obtained in this study
		*Praomys*	*Praomys rostratus*	Sequence obtained in this study
	Rattini	*Bandicota*	*Bandicota bengalensis*	Sequence obtained in this study
		*Berylmys*	*Berylmys bowersi*	Sequence obtained in this study
		*Bunomys*	*Bunomys chrysocomus*	Sequence obtained in this study
		*Chryromyscus*	*Chriromyscus chiropus*	Sequence obtained in this study
		*Diplothrix*	*Diplothrix legata*	Sequence obtained in this study
		*Leopoldamys*	*Leopoldamys edwardsi*	Sequence obtained in this study
		*Maxomys*	*Maxomys surifer*	Sequence obtained in this study
		*Micromys*	*Micromys minutus*	OZ004784
		*Niviventer*	*Niviventer confucianus*	Sequence obtained in this study
		*Rattus*	*Rattus exulans*	Sequence obtained in this study
			*Rattus norvegicus*	Rnor_6.0 (chromosome 2)
			*Rattus rattus*	NC_046156
			*Rattus tanezumi*	Sequence obtained in this study
Deomyinae		*Acomys*	*Acomys cahirinus*	CM057038
			*Acomys russatus*	LR87723 *
Gerbillinae		*Meriones*	*Meriones unguiculatus*	XM_021658779 *
		*Pachyuromys*	*Pachyuromys duprasi*	CM053744
		*Psammomys*	*Psammomys obesus*	XM_055628096
		*Rhombomys*	*Rhombomys opimus*	REGO01000051

The *Ovgp1* sequences were either retrieved from GenBank and Ensembl or were obtained in this study. The sequences used to determine the primers are indicated by an asterisk (*).

**Table 2:** Stop codon positions in the amplified sequences of *Ovgp1* (exons 1–6). The stop codon position is based on the alignment shown in Figure 1.

Gender/Species	Stop Codon Position
*Rattus norvegicus*	14, 20, 56, 59, 173, 200
*Rattus exulans*	28, 196, 250, 494
*Rattus rattus*	28, 173, 179, 200, 368, 380, 417, 426, 470, 498, 519, 605
*Rattus tanezumi*	28, 173, 179, 196, 250, 400
*Bandicota bengalensis*	196, 478
*Diplothrix legata*	196, 478
*Bunomys chrysocomus*	196, 478
*Berylmys bowersi*	56, 59, 179, 200, 494
*Niviventer confucianus*	478
*Chriromyscus chiropus*	178

**Table 3:** Peptides corresponding to chitinases (GH18 family) detected by HPLC-MS/MS in rat OF from oviducts with oocytes.

Protein (*Gene*)	Accession Number	Peptides	Sequence	Score	SPI	*m*/*z*	*n*
Chitinase domain-containing protein 1 (*Chid1*)	A0A0G2K3D1 ^1^ A0A0G2JSR1 ^2^ A0A140TAD5 ^3^	LALVCGSVH ^1,2,3^	10–18 ^1^ 13–21 ^2,3^	6.02	67.1	898.481	1
		TDIKAEDVVLEHRSYCSARARERNFAGEVLGYVTPWNSHGYDVAKVFGS ^1,2,3^	53–101 ^1^ 56–104 ^2,3^	7.64	73.8	5499.693	1
		VLEHRSYCSARAR ^1,2,3^	61–73 ^1^ 64–76 ^2,3^	6.91	73.6	1547.786	1
		ITGLHDVD ^1,2,3^	123–130 ^1^ 126–133 ^2,3^	5.89	64.6	869.436	1
		IHMLTHLAEALHQAR ^1,2,3^	206–220 ^1^ 209–223 ^2,3^	8.62	81.9	1740.933	4
		ILLGL ^1,2,3^	355–359 ^1^ 298–302 ^2^ 264–268 ^3^	5.02	80.2	528.376	1
		GMDYAASKDAREPVIGAR ^1,2,3^	363–380 ^1^ 306–323 ^2^ 272–289 ^3^	5.3	85.4	1906.944	1
		DAREPVIGA ^1,2,3^	371–379 ^1^ 314–322 ^2^ 280–288 ^3^	5.01	67.9	927.489	1
	A0A0G2K3D1 ^1^ A0A140TAD5 ^3^	IWELG ^1,3^	527–531 ^1^ 348–352 ^3^	5.28	62.9	617.329	1
	A0A0G2K3D1 ^1^	LLPTVPSLRAQ ^1^	457–467 ^1^	7.12	73.2	1194.72	10
	A0A0G2JSR1^2^	WILVS^2^	396–400	6.22	63.1	617.366	1
Chitinase-3-like protein 1 (*Chi3l1*)	A0A8I5ZNV1 ^1^ A4LA56 ^2^	LLSAAVSAGKV ^1,2^	190–200 ^1^ 152–162 ^2^	5.81	82.6	1015.615	5
		DRFSNVDYGVGYMLRL ^1,2^	249–264 ^1^ 211–226 ^2^	5.41	79.6	1904.932	1
		LVMGIPTFGK ^1,2^	271–280 ^1^ 233–242 ^2^	5.3	71.5	1062.602	1
		LVMGIPTFGKS ^1,2^	271–281 ^1^ 233–243 ^2^	6.02	67.3	1149.634	11
		KNKVKYLK ^1,2^	352–359 ^1^ 314–321 ^2^	5.9	60.5	1020.656	1
		KVKYLKNK ^1,2^	354–361 ^1^ 316–323 ^2^	5.58	75.7	1020.656	1
	A0A8I5ZNV1 ^1^	PGLTLDFPTGFAVLMLLQSCSAYKLVCYYTN ^1^	18–48 ^1^	5.04	91.3	3428.698	1
		TLDFPTGFAVL ^1^	21–31 ^1^	6.23	76.4	1180.625	3
		LSTSEWNDVTLYGMLNTLKTRLEHKRTRGGEDGQRFYPRFSRIVSNA ^1^	84–130 ^1^	6.89	71.2	5499.809	1
		NDVTLYGMLNTLKTRLEHK ^1^	90–108 ^1^	5.12	62.9	2246.196	1
		NDVTLYGMLNTLKTRLEHKRTRGGEDGQRFYPRFSR ^1^	90–125 ^1^	6.11	73.2	4212.227	1
		KTRLEHKRT ^1^	102–110 ^1^	7.16	64.9	1168.691	1
	A4LA56 ^2^	LVMGIPTFGKSFTLASSENQVGAPISGSGLPGRYTKEKGTLAYYEICDFLRG ^2^	233–284 ^2^	5.95	64.7	5526.808	1
Chitinase (*Chit1*)	F7ER89 ^1^ A0A8I6AV32 ^2^	SFLRTHGFDGLDLDW ^1,2^	118–132 ^1^ 125–139 ^2^	5.95	68.4	1778.85	2
		INLMAYDFHSSWDKTT ^1,2^	200–215 ^1^ 207–222 ^2^	5.46	81.4	1928.885	2
		AYDFHSSWDKTTG ^1,2^	204–216 ^1^ 211–223 ^2^	6.42	75.9	1514.655	2
		AEKNVDAAVTLWLQKGTPASKLMLGMPAYGRSFTLASSSDSGVGAPATGPGAPGPY ^1,2^	232–287 ^1^ 239–294 ^2^	7.04	71.2	5548.788	1
	F7ER89 ^1^	LVMRALALV ^1^	3–11 ^1^	5.4	81.8	985.623	1
		LALVGSAAK ^1^	8–16 ^1^	6.1	83.3	829.514	2
		LVGSAAKLFCY ^1^	10–20 ^1^	5.56	82.8	1171.618	3
		TEKSSFYSCGGGRLFQH ^1^	423–439 ^1^	5.14	63.5	1903.876	2
		LVFIDSCKCC ^1^	445–454 ^1^	5.96	82.4	1130.504	3
	A0A8I6AV32 ^2^	APQAWCLSTLANAVP ^2^	370–384 ^1^	5.5	100	1541.778	1
Acidic mammalian chitinase (*Chia*)	M9YP04 ^1^ A0A0G2K676 ^2^ A0A8I5Y1C5 ^3^ F1LPK5 ^4^	VIKFLRQYG ^1,2,3,4^	15–23 ^1,4^ 160–168 ^2^ 123–131 ^3^	6.33	78.5	1123.662	3
		LDLDWEYPGSRGSPPQDK ^1,2,3,4^	27–44 ^1,4^ 172–189 ^2^ 135–152	6.15	63.7	2059.972	4
		LDLDWEYPGSRGSPPQDKHLF ^1,2,3,4^	27–47 ^1,4^ 172–192 ^2^ 135–155 ^3^	5.82	60.4	2457.183	2
		IWAID ^1,2,3,4^	251–255 ^1^ 396–400 ^2^ 341–345 ^3^ 245–249 ^4^	5.28	62.9	617329	1
	M9YP04 ^1^	DVDYVMNYWKDNGAPAEKLIVGFPEYGHTYILSNPSDTG ^1^	134–172 ^1^	7.43	91.9	4376.049	2
		MIWAIDLDDFTGSFCDQGKFPLTSTLNKALDIPTADCTAPDLPSEPVTTPPG ^1^	250–301 ^1^	6.05	64.4	5522.637	1
	A0A0G2K676 ^2^	LETLVITRHSGGIK ^2^	15–24 ^2^	5.25	61.3	1523.89	1

Score: score based primarily on signal intensity. SPI: percentage of detected signals of each peptide fragment with respect to the theoretical number of signals that it should produce. *m*/*z*: mass/charge ratio of the detected fragment. “*n*” represents the number of times that the peptide was detected. In proteins with several sequences, the sequence(s) to which each peptide belongs is/are indicated as a superscript.

**Table 4:** Peptides corresponding to members of the GH18 family detected by HPLC-MS/MS in rat OF on day 3 post-fertilization.

Protein (*Gene*)	Accession Number	Peptides	Sequence	Score	SPI	*m*/*z*	*n*
Chitinase domain-containing protein 1 (*Chid1*)	A0A0G2K3D1 ^1^ A0A0G2JSR1 ^2^ A0A140TAD5 ^3^	VLWLALVCGSV ^1,2,3^	7–17 ^1^ 10–20 ^2,3^	6.52	63.9	580.3357	2
		VILVI ^1,2,3^	223–227 ^1^ 226–230 ^2,3^	5.41	87	556.4055	3
	A0A0G2K3D1 ^1^ A0A140TAD5 ^3^	IWELGQGLDYFY ^1,3^	527–538 ^1^ 348–359 ^3^	5.92	86.1	501.9137	1
	A0A0G2K3D1 ^1^	AVTPGPLEGIDEYSSRLST ^1^	230–248 ^1^	6.7	66	664.6733	1
		IQLSKSTACPNIAFVGI ^1^	381–397 ^1^	6.12	69	633.6712	1
		LLPTVPSLRAQ ^1^	457–467 ^1^	8.04	70.1	637.8669	15
	A0A0G2JSR1 ^2^	VALPLAVSSQQIWTLGRG ^2^	328–345 ^2^	6.45	100	659.3501	1
		LGRGGSTSALLLAGLGLAS ^2^	342–360 ^2^	5.35	72.4	572.0094	1
	A0A140TAD5 ^3^	QWRSKILLGLNFYGMDYAASKDAREPVIGARYIQTLK ^3^	259–295 ^3^	5.31	100	883.8151	1
Chitinase-3-like protein 1 (*Chi3l1*)	A0A8I5ZNV1 ^1^ A4LA56 ^2^	LLSAAVSAGKV ^1,2^	190–200 ^1^ 152–162 ^2^	8.58	90.5	548.3063	4
		VAQIAQHLDFINLMTYD ^1,2^	208–224 ^1^ 170–186 ^2^	6.82	74.6	664.6733	1
		LVMGIPTFGK ^1,2^	271–280 ^1^ 233–242 ^2^	5.46	72.9	531.7895	1
		LVMGIPTFGKS ^1,2^	271–281 ^1^ 233–243 ^2^	7.44	66.7	575.3115	10
	A0A8I5ZNV1 ^1^	VTLYGMLNTLKTRLE ^1^	92–106 ^1^	5.95	69.9	611.3092	2
		TGSGLPGRYTKEKGTLA ^1^	296–312 ^1^	5.76	65.8	579.2939	1
	A4LA56 ^2^	ISGSGLPGRYTKEK ^2^	257–270 ^2^	5.14	73.4	524.9144	1
Chitinase (*Chit1*)	F7ER89 ^1^ A0A8I6AV32 ^2^	VDPNLCTHVIYAFAGLN ^1,2^	39–55 ^1^ 46–62 ^2^	7.8	62.3	952.4638	1
		VSTVEPNDELFYQELNS ^1,2^	59–75 ^1^ 66–82 ^2^	5.65	67.7	1032.472	1
		SFLRTHGFDGLDLDW ^1,2^	118–132 ^1^ 125–139 ^2^	5.5	64.6	889.9392	1
		DAAVTLWLQK ^1,2^	237–246 ^1^ 244–253 ^2^	5.59	65	572.8127	1
	F7ER89 ^1^	LVMRALALV ^1^	3–11 ^1^	6.12	78.7	501.3022	1
		LVMRALALVGSAA ^1^	3–15 ^1^	5.33	73.7	456.593	1
		LALVGSAAK ^1^	8–16 ^1^	5.71	70.2	415.2688	2
		LVGSAAKLFCY ^1^	10–20 ^1^	7.83	60.9	586.2993	1
		LVFIDSCKCC ^1^	445–454 ^1^	7.22	72	396.5257	4
Acidic mammalian chitinase (*Chia*)	M9YP04 ^1^ A0A0G2K676 ^2^ A0A8I5Y1C5 ^3^ F1LPK5 ^4^	VIKFLRQYG ^1,2,3,4^	15–23 ^1,4^ 160–168 ^2^ 123–131 ^3^	6.39	88.7	602.3328	6
		LDLDWEYPGSRGSPPQDK ^1,2,3,4^	27–44 ^1,4^ 172–189 ^2^ 135–152 ^3^	5.83	70.6	687.3472	18
	A0A0G2K676 ^2^ A0A8I5Y1C5 ^3^ F1LPK5 ^4^	PYAYKGNEWVGYDNIKS^2,3,4^	362–378 ^2^ 307–323 ^3^ 211–227 ^4^	5.51	75	668.6433	2
	A0A0G2K676 ^2^ A0A8I5Y1C5 ^3^	LVCYFTNWAQYR ^2,3^	61–72 ^2^ 24–35 ^3^	5.7	100	567.6036	2
		IYAFAGMQNNQITTI ^2,3^	92–106 ^2^ 55–69 ^3^	5.78	60.5	562.2797	3
		FAGMQNNQITT ^2,3^	95–105 ^2^ 58–68 ^3^	5.1	66.6	435.5326	2
	A0A0G2K676 ^2^	ITRHSGGIK ^2^	16–24 ^2^	7.1	77.6	564.7709	2
	A0A8I5Y1C5 ^3^	AVAAGISNIQAAL ^3^	183–195 ^3^	5.49	79.2	400.2401	1
		IVSLPNSPLYKL ^3^	202–213 ^3^	5.6	76.4	501.9132	1
	F1LPK5 ^4^	VAAALYLILRCIVYLDF ^4^	76–92 ^4^	5.92	100	652.6861	2
		VYLDFIHVMTYDLHGS ^4^	88–103 ^4^	5.69	67.1	1043.4592	1

Score: score based primarily on signal intensity. SPI: percentage of detected signals of each peptide fragment with respect to the theoretical number of signals that it should produce. *m*/*z*: mass/charge ratio of the detected fragment. “*n*” represents the number of times that the peptide was detected. In proteins with several sequences, the sequence(s) to which each peptide belongs is/are indicated as a superscript.

## Data Availability

Data are contained within the article and Supplementary Materials.

## Supplementary Materials

The following supporting information can be downloaded at: https://www.mdpi.com/article/10.3390/ani15010055/s1. Table S1. Primers used for the amplification of the Ovgp1 gDNA (exons 1 to 6). Figure S1. Primer location in the mouse Ovgp1 gDNA sequence (ENSMUSG00000074340). The size of exons 1 to 6, the amplicons generated by each primer pair and the microsatellites present in this fragment of the sequence are indicated. Exon 1 consists of a non-coding part represented in white and the part downstream of the initial methionine represented in grey which translates into protein. Table S2. Primers used in RT-qPCR. Figure S2. Multiple alignment of the non-Rattini murid species analysed without premature stop codons. The letter “N” in the nucleotide sequence and the letter “X” in the amino acid sequence appear when the base or amino acid, respectively, could not be determined. The letter “Y” in the nucleotide sequence represents that the nucleotide at that position may be either C or T. The presence of the symbol “-” indicates absence of sequence due to a lack of sequencing or an actual deletion.
