# Supplementary material for: Molecular Evolution of the Ovgp1 Gene in the Subfamily Murinae
Source: Animals (Basel). 2024 Dec 29;15(1):55. doi: 10.3390/ani15010055 (PMC11719014; doi:10.3390/ani15010055)
Supplement: Supplementary file 1 [file animals-15-00055-s001.zip › animals-3352160-supplementary.pdf]

Supplementary Materials:

Table S1. Primers used for the amplification of the *Ovgp1* gDNA (exons 1 to 6).

Name	Pb (d/r)	Sequence (5'→3')	Tm (°C)
**Fw1**	19 (d)	CATAGCTACCAGGCAGTTG	58
**Rv1**	21 (r)	CTTCATCTTGGACCAGTTAAT	58
**Rv1b**	21 (r)	ATTAACTGGTCCAAGATGAAG	58
**Fw2**	21 (d)	CTTCATCTTGGACCAGTTAAT	58
**Rv2**	20 (r)	GAGGCAAAGGCAAATATCAG	58
**Fw3**	20 (d)	ACTGCCTACAAACTGGTGTG	60
**Rv3**	19 (r)	GGCAGTTGCAAGAGGCAAG	60
**Fw4**	21 (d)	CAGAGTTCAACAAACTCAAGG	60
**Rv4**	20 (r)	CTGGGAGGTAGAGCAAGATG	58
**Rv4b**	20 (r)	CATCTTGCTCTACCTCCCAG	58
**Fw5**	21 (d)	AGTGACTTAAGGTGTCCATAG	60
**Rv5**	23 (r)	CTAAGTTTCACATCAGATGTCTA	62
**Fw6**	19 (d)	CTTGTGCCAACCAAGCTTC	62
**Rv6**	20 (r)	GGCAAACTGGAGCTCCTAAG	62
**Fw7**	20 (d)	TGTCTTTCTCATAGACTTGG	56
**Rv7**	19 (r)	GTTACCTGGCAACACAACA	56

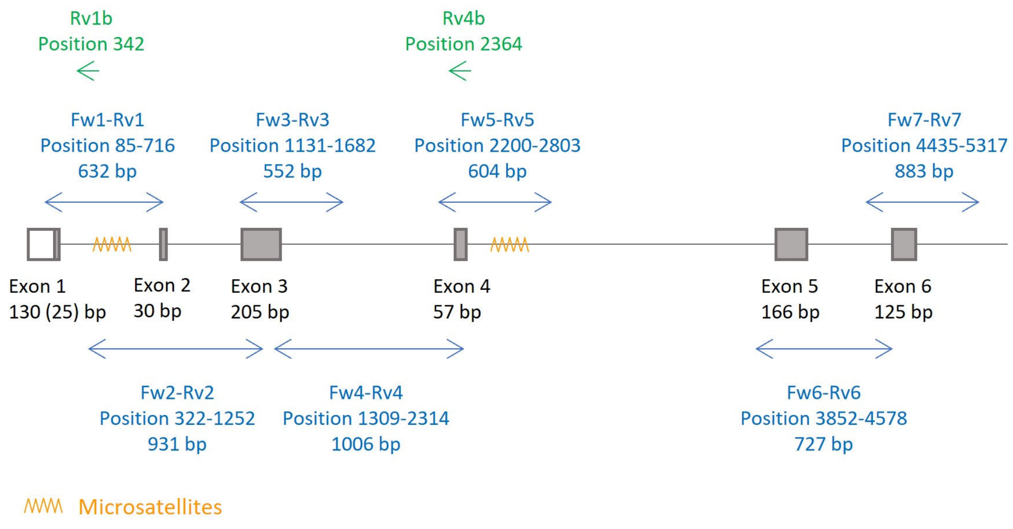

Figure S1. Primer location in the mouse *Ovgp1* gDNA sequence (ENSMUSG00000074340). The size of exons 1 to 6, the amplicons generated by each primer pair and the microsatellites present in this fragment of the sequence are indicated. Exon 1 consists of a non-coding part represented in white and the part downstream of the initial methionine represented in grey which translates into protein.

Table S2. Primers used in RT-qPCR.

Name	Pb (d/r)	Sequence (5'→3')	Tm (°C)
**Fw-*Chia***	23 (d)	GAACTTTGGAACTGCTCCTTTCA	59
**Rv-*Chia***	20 (r)	GTCCAGGTCCAGTCCATCAA	59
**Fw-*Chit1***	20 (d)	AGAGCCCAATGACGAGCTTT	59
**Rv-*Chit1***	21 (r)	TTGAGCTTGGGGTTCCTCTTC	59
**Fw-*Chi3l1***	20 (d)	TTGCCCAGATAGCCCAACAC	60
**Rv-*Chi3l1***	20 (r)	GAAGAGGGGGCTGTGATGTC	60
**Fw-*Chid1***	23 (d)	TGCGAGCTGTCAAGAAGCAT	59
**Rv-*Chid1***	20 (r)	AACAGAAGCCGAGGCACTAT	59
**Fw-*Actb1***		pre-designed primers	

	(Merck KGaA, Germany)
**Rv-** ***Actb1***	pre-designed primers (Merck KGaA, Germany)
**Fw-** ***Hprt1***	pre-designed primers (Merck KGaA, Germany)
**Rv-** ***Hprt1***	pre-designed primers (Merck KGaA, Germany)

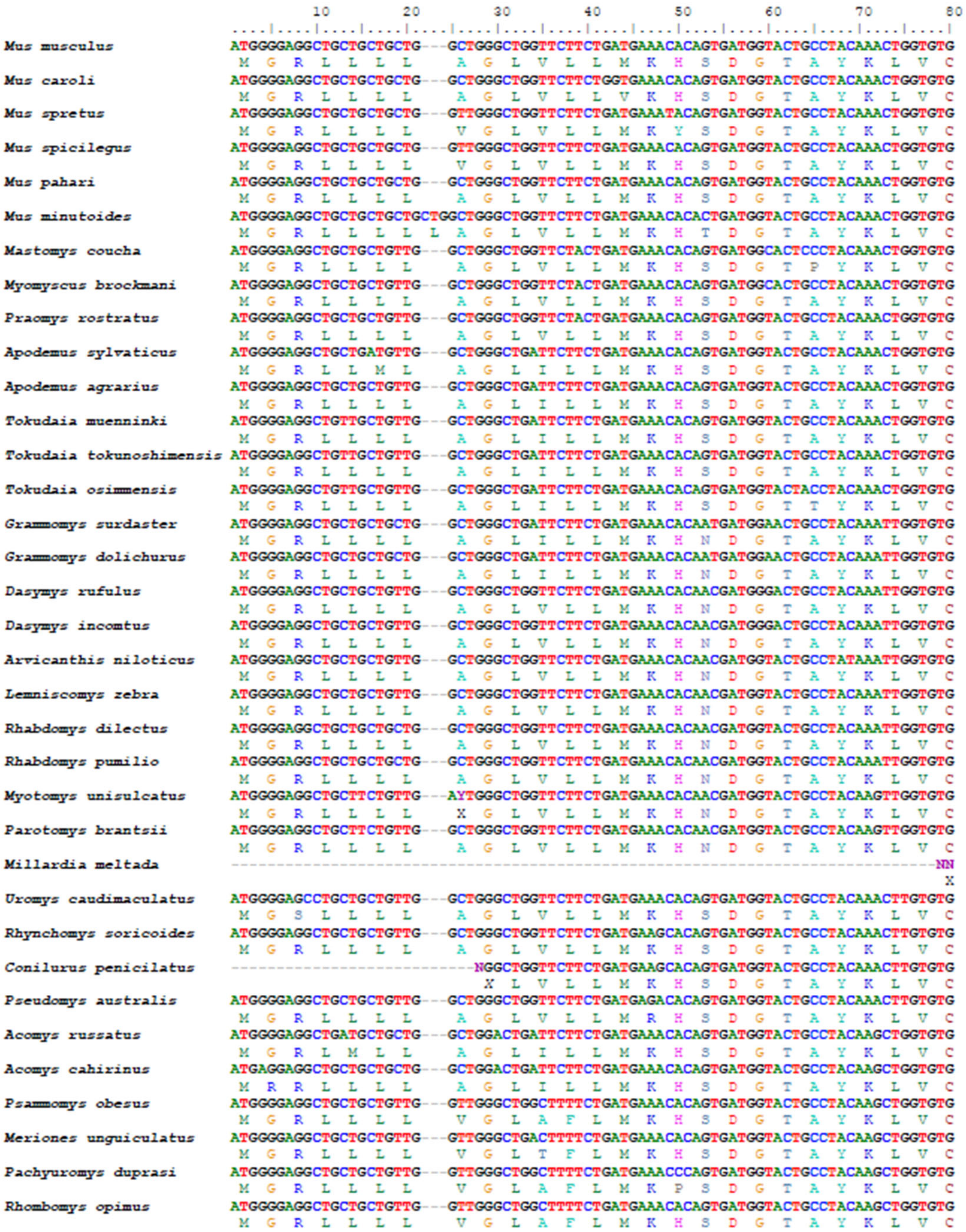

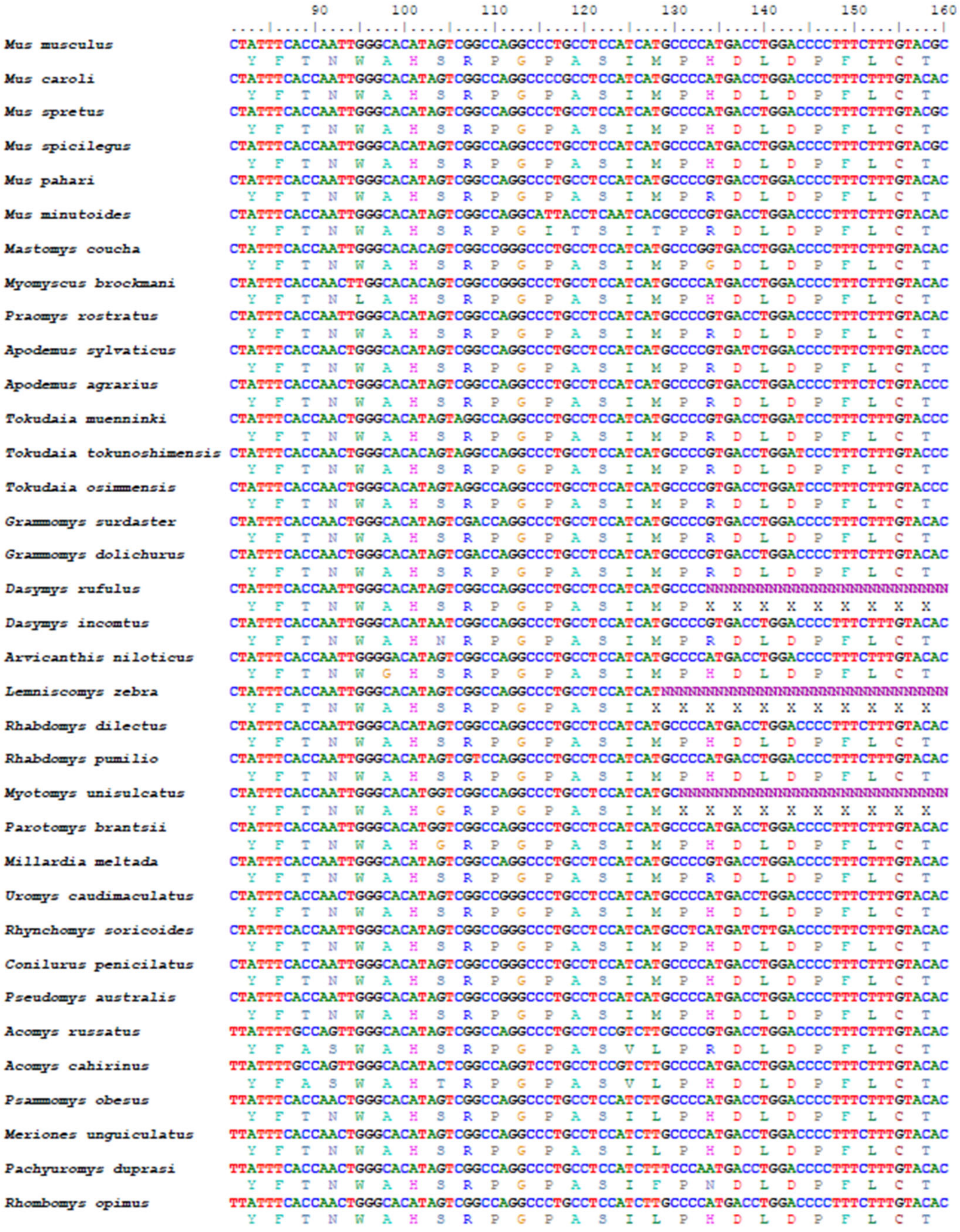
90
100
110
120
130
140
150
160
Mus musculus
Mus caroli
Mus spretus
Mus spicilegus
Mus pahari
Mus minutoides
Mastomys coucha
Myomyscus brockmani
Praomys rostratus
Apodemus sylvaticus
Apodemus agrarius
Tokudaia muenninki
Tokudaia tokunoshimensis
Tokudaia osimensis
Grammomys surdaster
Grammomys dolichurus
Dasymys rufulus
Dasymys incomtus
Arvicanthis niloticus
Lemniscomys zebra
Rhabdomys dilectus
Rhabdomys pumilio
Myotomys unisulcatus
Parotomys brantsii
Millardia meltada
Uromys caudimaculatus
Rhynchomys soricoides
Conilurus penicilatus
Pseudomys australis
Acomys russatus
Acomys cahirinus
Psammomys obesus
Meriones unguiculatus
Pachyuromys duprasi
Rhombomys opimus

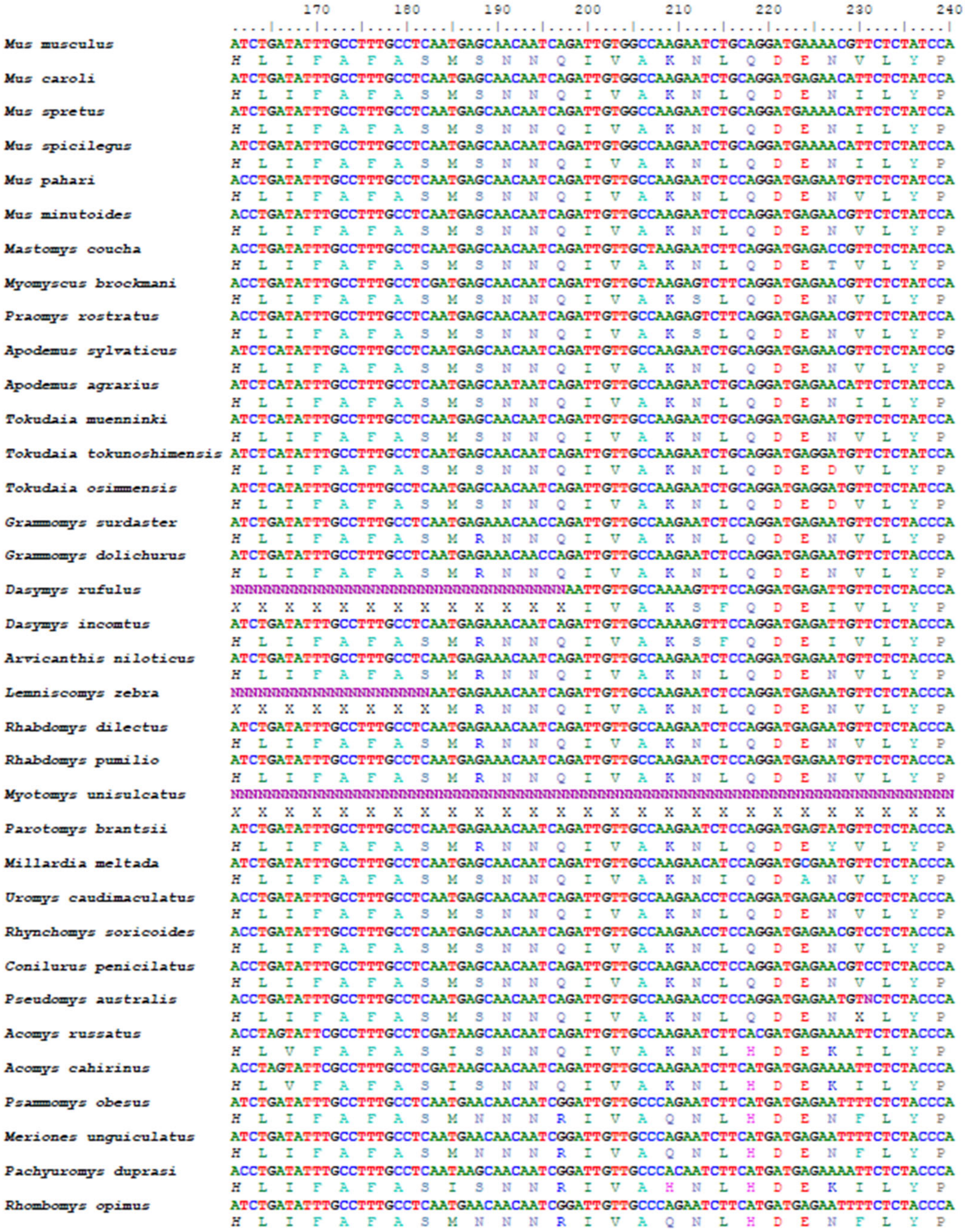
170 180 190 200 210 220 230 240
Mus musculus
Mus caroli
Mus spretus
Mus spicilegus
Mus pahari
Mus minutoides
Mastomys coucha
Myomyscus brockmani
Praomys rostratus
Apodemus sylvaticus
Apodemus agrarius
Tokudaia muenninki
Tokudaia tokunoshimensis
Tokudaia osimensis
Grammomys surdaster
Grammomys dolichurus
Dasymys rufulus
Dasymys incomtus
Arvicanthis niloticus
Lemniscomys zebra
Rhabdomys dilectus
Rhabdomys pumilio
Myotomys unisulcatus
Parotomys brantsii
Millardia meltada
Uromys caudimaculatus
Rhynchomys soricoides
Conilurus penicilatus
Pseudomys australis
Acomys russatus
Acomys cahirinus
Psammomys obesus
Meriones unguiculatus
Pachyuromys duprasi
Rhombomys opimus

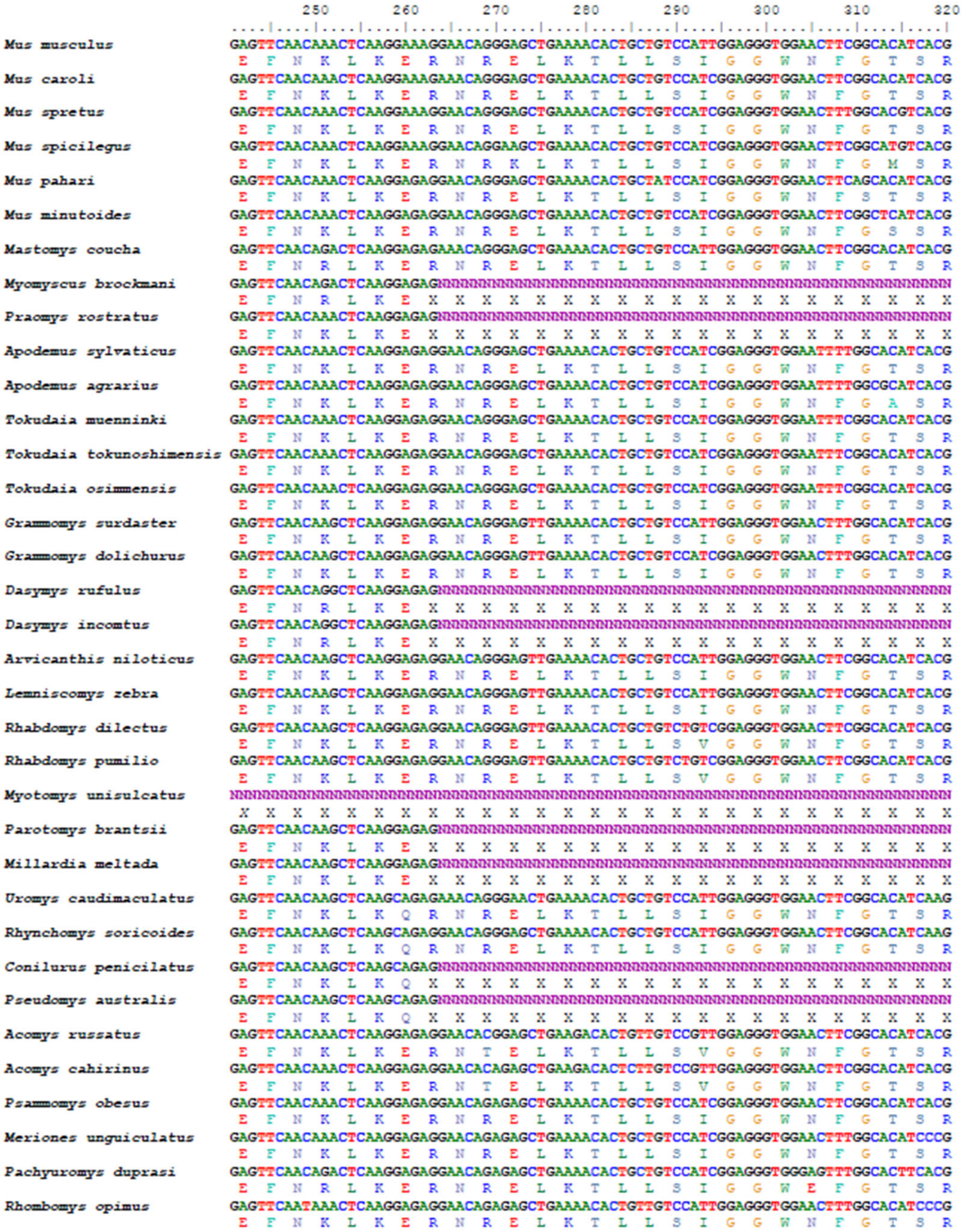

250 260 270 280 290 300 310 320
Mus musculus
Mus caroli
Mus spretus
Mus spicilegus
Mus pahari
Mus minutoides
Mastomys coucha
Myomyscus brockmani
Praomys rostratus
Apodemus sylvaticus
Apodemus agrarius
Tokudaia muenninki
Tokudaia tokunoshimensis
Tokudaia osimensis
Grammomys surdaster
Grammomys dolichurus
Dasymys rufulus
Dasymys incomtus
Arvicanthis niloticus
Lemniscomys zebra
Rhabdomys dilectus
Rhabdomys pumilio
Myotomys unisulcatus
Parotomys brantsii
Millardia meltada
Uromys caudimaculatus
Rhynchomys soricoides
Conilurus penicilatus
Pseudomys australis
Acomys russatus
Acomys cahirinus
Psammomys obesus
Meriones unguiculatus
Pachyuromys duprasi
Rhombomys opimus

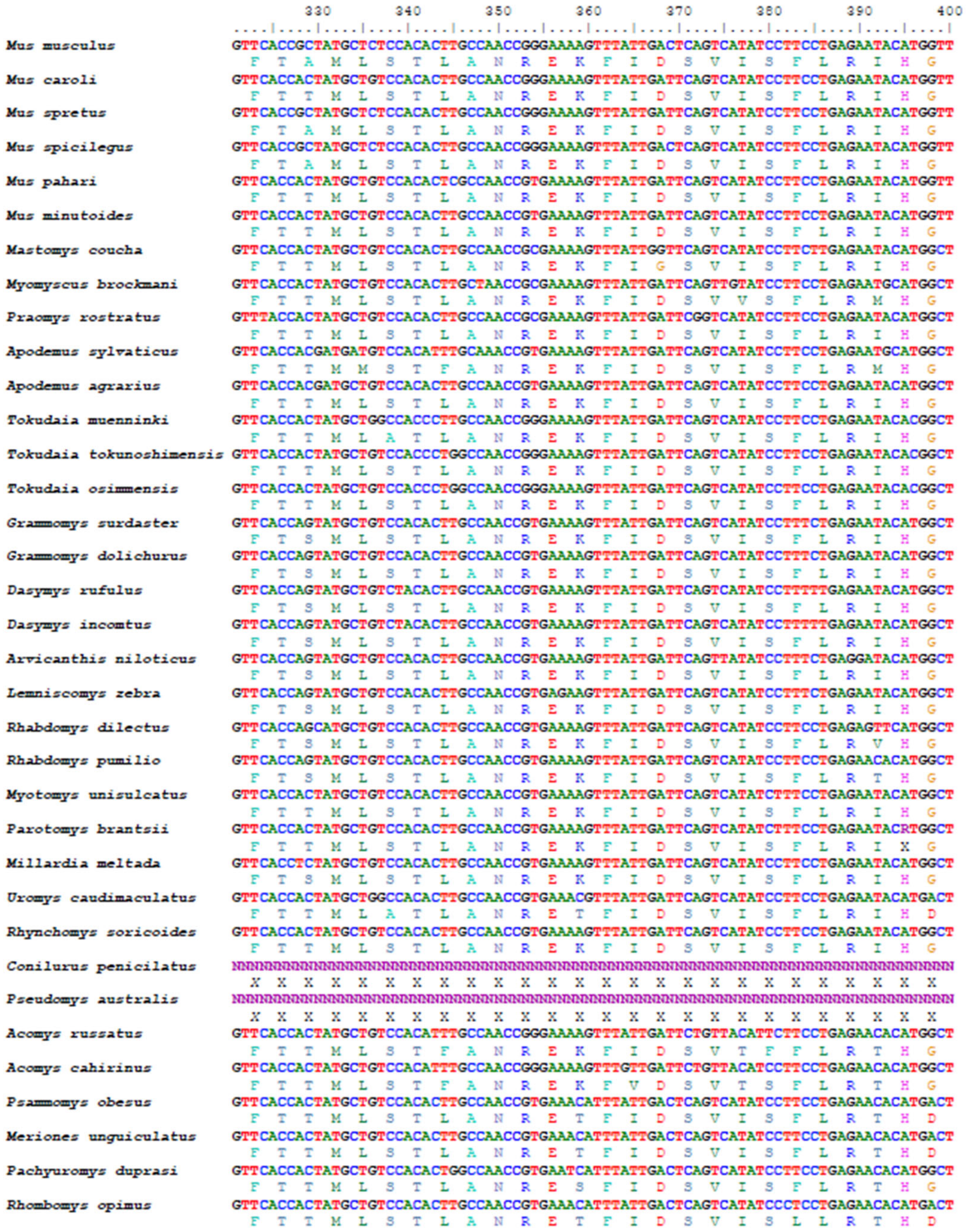
330
340
350
360
370
380
390
400
Mus musculus
Mus caroli
Mus spretus
Mus spicilegus
Mus pahari
Mus minutoides
Mastomys coucha
Myomyscus brockmani
Praomys rostratus
Apodemus sylvaticus
Apodemus agrarius
Tokudaia muenninki
Tokudaia tokunoshimensis
Tokudaia osimensis
Grammomys surdaster
Grammomys dolichurus
Dasymys rufulus
Dasymys incomtus
Arvicanthis niloticus
Lemniscomys zebra
Rhabdomys dilectus
Rhabdomys pumilio
Myotomys unisulcatus
Parotomys brantsii
Millardia meltada
Uromys caudimaculatus
Rhynchomys soricoides
Conilurus penicilatus
Pseudomys australis
Acomys russatus
Acomys cahirinus
Psammomys obesus
Meriones unguiculatus
Pachyuromys duprasi
Rhombomys opimus

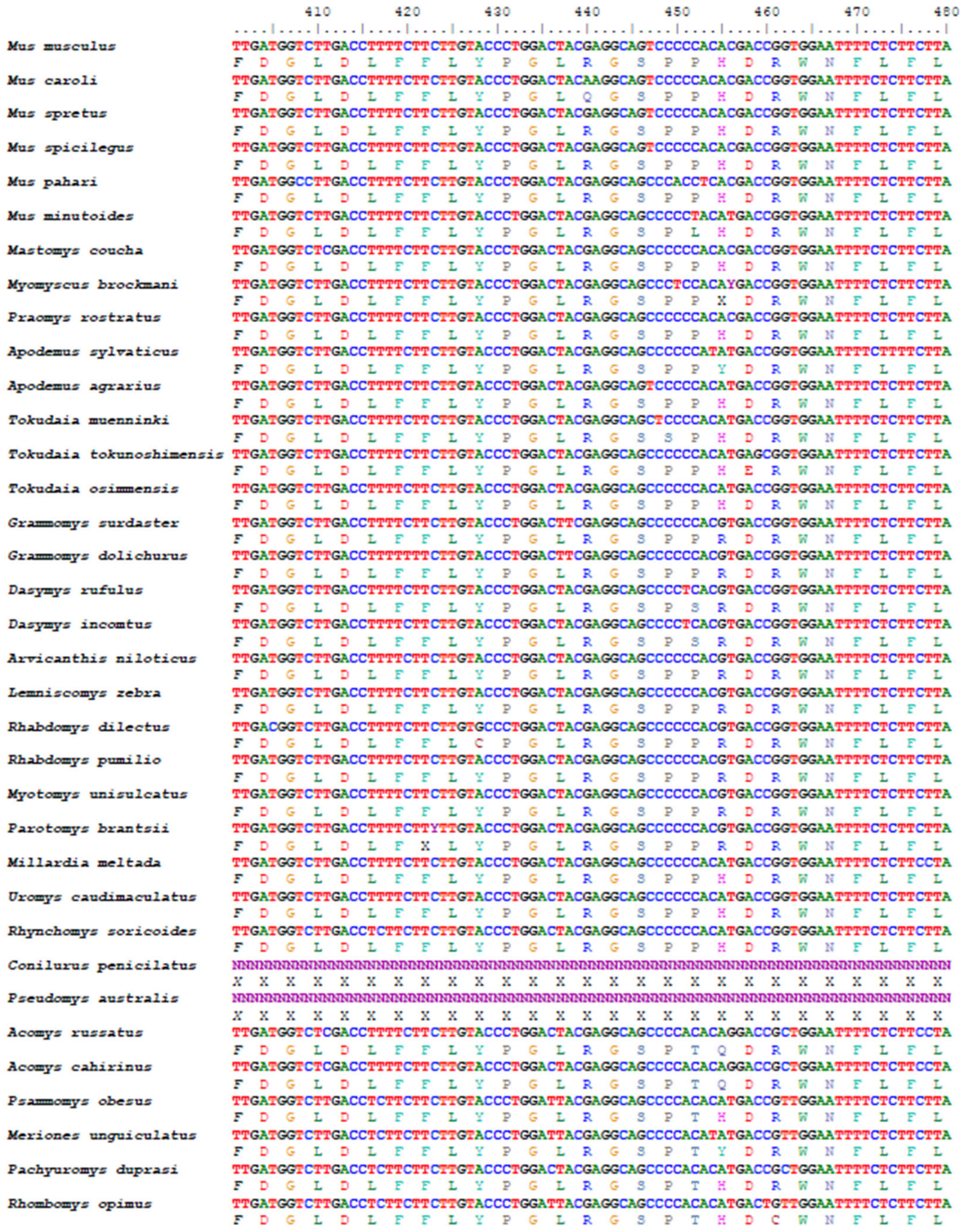
410 420 430 440 450 460 470 480
Mus musculus
Mus caroli
Mus spretus
Mus spicilegus
Mus pahari
Mus minutoides
Mastomys coucha
Myomyscus brockmani
Praomys rostratus
Apodemus sylvaticus
Apodemus agrarius
Tokudaia muenninki
Tokudaia tokunoshimensis
Tokudaia osimensis
Grammomys surdaster
Grammomys dolichurus
Dasymys rufulus
Dasymys incomtus
Arvicanthis niloticus
Lemniscomys zebra
Rhabdomys dilectus
Rhabdomys pumilio
Myotomys unisulcatus
Parotomys brantsii
Millardia meltada
Uromys caudimaculatus
Rhynchomys soricoides
Conilurus penicilatus
Pseudomys australis
Acomys russatus
Acomys cahirinus
Psammomys obesus
Meriones unguiculatus
Pachyuromys duprasi
Rhombomys opimus

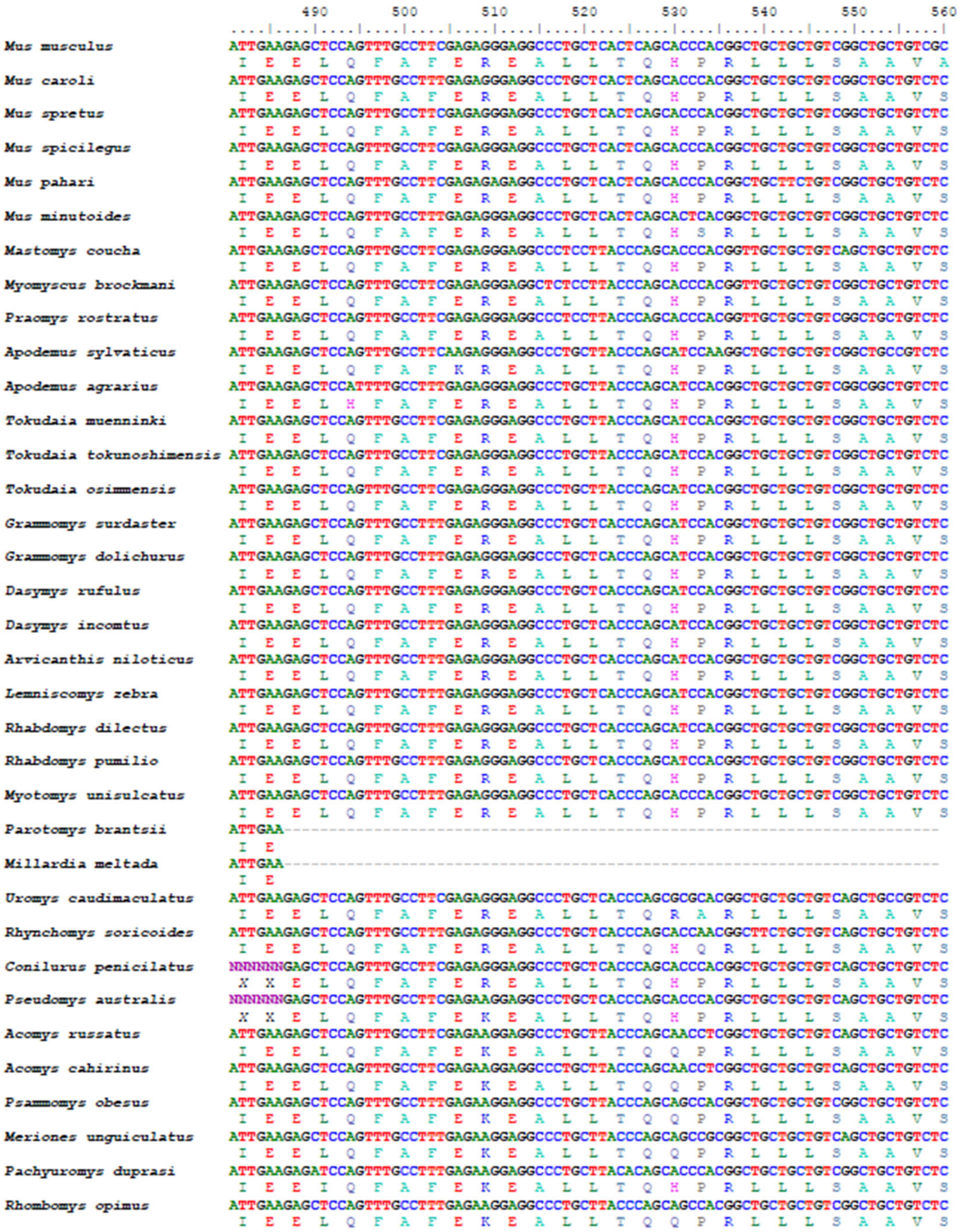
490
500
510
520
530
540
550
560
Mus musculus
Mus caroli
Mus spretus
Mus spicilegus
Mus pahari
Mus minutoides
Mastomys coucha
Myomyscus brockmani
Praomys rostratus
Apodemus sylvaticus
Apodemus agrarius
Tokudaia muenninki
Tokudaia tokunoshimensis
Tokudaia osimensis
Grammomys surdaster
Grammomys dolichurus
Dasymys rufulus
Dasymys incomtus
Arvicanthis niloticus
Lemniscomys zebra
Rhabdomys dilectus
Rhabdomys pumilio
Myotomys unisulcatus
Parotomys brantsii
Millardia meltada
Uromys caudimaculatus
Rhynchomys soricoides
Conilurus penicilatus
Pseudomys australis
Acomys russatus
Acomys cahirinus
Psammomys obesus
Meriones unguiculatus
Pachyuromys duprasi
Rhombomys opimus

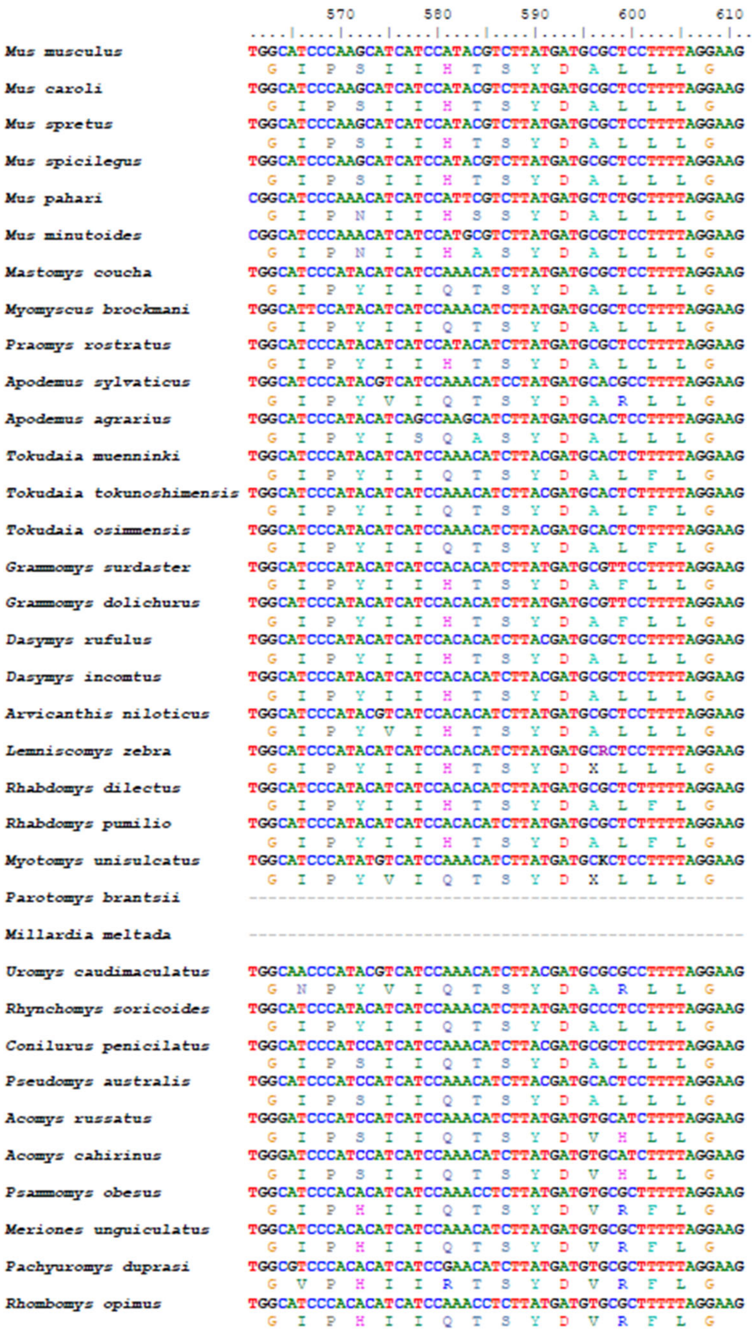

Figure S2. Multiple alignment of the non-Rattini murid species analysed without premature stop codons. The letter "N" in the nucleotide sequence and the letter "X" in the amino acid sequence appear when the base or amino acid, respectively, could not be determined. The letter "Y" in the nucleotide sequence represents that the nucleotide at that position may be either C or T. The presence of the symbol "-" indicates absence of sequence due to a lack of sequencing or an actual deletion.
